# *catena*-Poly[[[tetra­aqua­iron(II)]-μ-5,5′-diazenediylditetra­zolido] dihydrate]

**DOI:** 10.1107/S1600536810039632

**Published:** 2010-10-09

**Authors:** Bao-juan Jiao, Zhi-jun Yan, Guang Fan, San-ping Chen, Sheng-li Gao

**Affiliations:** a Department of Chemistry and Chemical Engineering, Xi’an University of Arts & Science, Xi’an 710065, Shaanxi, People’s Republic of China; b College of Chemistry and Chemical Engineering, Xianyang Normal University, Xianyang 712000, Shaanxi, People’s Republic of China; c College of Chemistry and Materials Science, Northwest University, Xi’an 710069, Shaanxi, People’s Republic of China

## Abstract

In the title compound, {[Fe(C_2_N_10_)(H_2_O)_4_]·2H_2_O}_*n*_, the coordin­ation geometry around the Fe(II) atom, which lies on a center of inversion, is distorted octa­hedral, with bonds to four O atoms and two N atoms. The azotetra­zolate ligand displays a bridging coordination mode, forming an infinite zigzag chain. Inter­molecular O—H⋯O and O—H⋯N hydrogen bonding and offset face-to-face π–π stacking inter­actions [centroid–centroid distance = 3.4738 (13) Å] lead to a three-dimensional network.

## Related literature

For energetic complexes, see: Hammerl *et al.* (2001 ▶, 2002 ▶); Jiao *et al.* (2007 ▶).
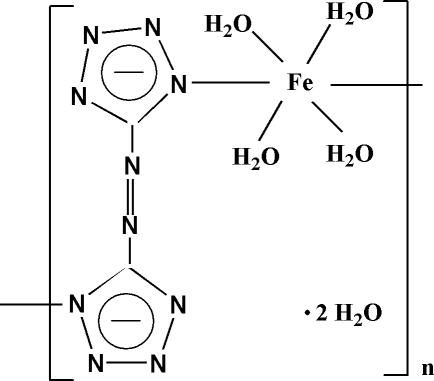


## Experimental

### 

#### Crystal data

[Fe(C_2_N_10_)(H_2_O)_4_]·2H_2_O
*M* *_r_* = 328.07
Triclinic, 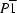

*a* = 6.2449 (5) Å
*b* = 6.9764 (6) Å
*c* = 7.8256 (6) Å
α = 76.424 (1)°
β = 74.135 (1)°
γ = 69.844 (1)°
*V* = 304.11 (4) Å^3^
*Z* = 1
Mo *K*α radiation
μ = 1.29 mm^−1^
*T* = 273 K
0.30 × 0.18 × 0.12 mm

#### Data collection

Bruker SMART APEX CCD diffractometer
Absorption correction: multi-scan (*SADABS*; Bruker, 2002 ▶) *T*_min_ = 0.699, *T*_max_ = 0.861
1564 measured reflections
1061 independent reflections
973 reflections with *I* > 2σ(*I*)
*R*_int_ = 0.012

#### Refinement

*R*[*F* ^2^ > 2σ(*F* ^2^)] = 0.026
*wR*(*F* ^2^) = 0.070
*S* = 1.13
1061 reflections
106 parameters
6 restraints
H atoms treated by a mixture of independent and constrained refinement
Δρ_max_ = 0.24 e Å^−3^
Δρ_min_ = −0.35 e Å^−3^

### 

Data collection: *SMART* (Bruker, 2002 ▶); cell refinement: *SAINT* (Bruker, 2002 ▶); data reduction: *SAINT*; program(s) used to solve structure: *SHELXS97* (Sheldrick, 2008 ▶); program(s) used to refine structure: *SHELXL97* (Sheldrick, 2008 ▶); molecular graphics: *SHELXTL* (Sheldrick, 2008 ▶); software used to prepare material for publication: *SHELXL97*.

## Supplementary Material

Crystal structure: contains datablocks I, global. DOI: 10.1107/S1600536810039632/ng5036sup1.cif

Structure factors: contains datablocks I. DOI: 10.1107/S1600536810039632/ng5036Isup2.hkl

Additional supplementary materials:  crystallographic information; 3D view; checkCIF report

## Figures and Tables

**Table 1:** Hydrogen-bond geometry (Å, °)

*D*—H⋯*A*	*D*—H	H⋯*A*	*D*⋯*A*	*D*—H⋯*A*
O2—H2*A*⋯O3	0.85 (1)	1.86 (1)	2.699 (2)	170 (2)
O1—H1*A*⋯O3^i^	0.85 (1)	1.87 (1)	2.715 (2)	171 (3)
O1—H1*B*⋯N5	0.85 (1)	2.23 (2)	2.926 (2)	139 (2)
O3—H3*B*⋯N3^ii^	0.84 (1)	2.01 (1)	2.839 (2)	169 (2)
O3—H3*A*⋯N2^iii^	0.85 (1)	2.00 (1)	2.843 (2)	173 (2)
O2—H2*B*⋯N3^iv^	0.85 (1)	2.69 (2)	3.439 (2)	148 (2)
O2—H2*B*⋯N4^iv^	0.85 (1)	1.99 (1)	2.840 (2)	173 (2)

Symmetry codes: (i) 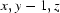; (ii) 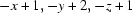; (iii) 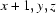; (iv) 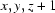.

**Fig. 1.**
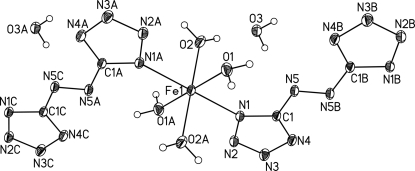
A view of the molecular structure of (I) with the atom-labling scheme. Displacement ellipsoids are drawn at the 30% probability level and H atoms are shown as small spheres of arbitrary radii.

**Fig. 2.**
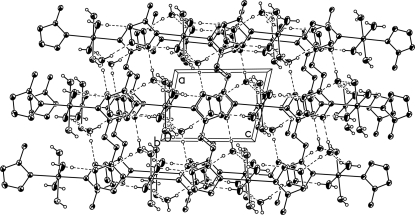
Three dimensional network of the title complex.

## supplementary crystallographic information

### Comment

After Thiele prepared metallic salts of azotetrazole and claimed these for use in initiators, salts of AT^2-^ (AT = 5,5'-azotetrazolate) have been extensively investigated and have often been considered for practical use as a class of energetic materials. We report here the crystal structure of the title compound, [Fe(C_2_N_10_)(H_2_0)_4_.2H_2_0]_n_, (I).

Single-crystal analysis shows that (I) exists as a one-dimensional infinite chain. As shown in Figure 1, the coordination geometry around Fe^2+^ cation can be described a disordered octahedral arrangement with coordination number of 6, where O1, O2, O1A and O2A form the equatorial plane, and axial positions are occupied by N1 and N1A. Additionally, each AT^2-^ provides two terminal nitrogen atoms (N1 and N1A) acting as bridging ligand to connect two [Fe(H_2_O)_4_]^2+^ to form an infinite zigzag chain.

In the crystal structure, the interactions of hydrogen bonding between the water molecules and the N atoms in the terazole rings, the off-set face to face π-π stacking interactions of the terazole rings link the complex to a three dimensional structure, as shown in Figure 2.

### Experimental

Brown block-like crystal for X-ray diffraction analysis was obtained from the mixture of (NH_4_)_2_Fe(SO4)_2_.6H_2_O (0.392 g, 1 mmol), Na_2_AT.5H_2_O (0.304 g,1 mmol) and distilled H_2_O (20 ml), which was allowed to evaporate at room temperature for one week.

### Refinement

H atoms attached to O atoms were placed in calculated positions, with O—H distances of 0.86 Å. The *U*_iso_(H) values were constrained to be -1.5Ueq of the carrier atom.

### Crystal data

**Table:**

[Fe(C_2_N_10_)(H_2_O)_4_]·2H_2_O	*Z* = 1
*M**_r_* = 328.07	*F*(000) = 168
Triclinic, *P*1	*D*_x_ = 1.791 Mg m^−^^3^
*a* = 6.2449 (5) Å	Mo *K*α radiation, λ = 0.71073 Å
*b* = 6.9764 (6) Å	Cell parameters from 1061 reflections
*c* = 7.8256 (6) Å	θ = 2.7–25.1°
α = 76.424 (1)°	µ = 1.29 mm^−^^1^
β = 74.135 (1)°	*T* = 273 K
γ = 69.844 (1)°	Block, brown
*V* = 304.11 (4) Å^3^	0.30 × 0.18 × 0.12 mm

### Data collection

**Table:**

Bruker SMART APEX CCD diffractometer	1061 independent reflections
Radiation source: fine-focus sealed tube	973 reflections with *I* > 2σ(*I*)
graphite	*R*_int_ = 0.012
φ and ω scans	θ_max_ = 25.1°, θ_min_ = 2.7°
Absorption correction: multi-scan (*SADABS*; Bruker, 2002)	*h* = −7→6
*T*_min_ = 0.699, *T*_max_ = 0.861	*k* = −7→8
1564 measured reflections	*l* = −8→9

### Refinement

**Table:**

Refinement on *F*^2^	Primary atom site location: structure-invariant direct methods
Least-squares matrix: full	Secondary atom site location: difference Fourier map
*R*[*F*^2^ > 2σ(*F*^2^)] = 0.026	Hydrogen site location: inferred from neighbouring sites
*wR*(*F*^2^) = 0.070	H atoms treated by a mixture of independent and constrained refinement
*S* = 1.13	*w* = 1/[σ^2^(*F*_o_^2^) + (0.0447*P*)^2^ + 0.0115*P*] where *P* = (*F*_o_^2^ + 2*F*_c_^2^)/3
1061 reflections	(Δ/σ)_max_ = 0.007
106 parameters	Δρ_max_ = 0.24 e Å^−^^3^
6 restraints	Δρ_min_ = −0.35 e Å^−^^3^

### Special details

**Table:**

Geometry. All e.s.d.'s (except the e.s.d. in the dihedral angle between two l.s. planes) are estimated using the full covariance matrix. The cell e.s.d.'s are taken into account individually in the estimation of e.s.d.'s in distances, angles and torsion angles; correlations between e.s.d.'s in cell parameters are only used when they are defined by crystal symmetry. An approximate (isotropic) treatment of cell e.s.d.'s is used for estimating e.s.d.'s involving l.s. planes.
Refinement. Refinement of *F*^2^ against ALL reflections. The weighted *R*-factor *wR* and goodness of fit *S* are based on *F*^2^, conventional *R*-factors *R* are based on *F*, with *F* set to zero for negative *F*^2^. The threshold expression of *F*^2^ > σ(*F*^2^) is used only for calculating *R*-factors(gt) *etc*. and is not relevant to the choice of reflections for refinement. *R*-factors based on *F*^2^ are statistically about twice as large as those based on *F*, and *R*- factors based on ALL data will be even larger.

### Fractional atomic coordinates and isotropic or equivalent isotropic displacement parameters (Å^2^)

**Table:**

	*x*	*y*	*z*	*U*_iso_*/*U*_eq_	
Fe1	0.5000	0.5000	1.0000	0.02375 (17)	
N1	0.5314 (3)	0.6482 (3)	0.7101 (2)	0.0240 (4)	
N2	0.3404 (3)	0.7685 (3)	0.6503 (2)	0.0287 (4)	
N3	0.3949 (3)	0.8147 (3)	0.4742 (2)	0.0323 (4)	
N4	0.6210 (3)	0.7259 (3)	0.4135 (2)	0.0287 (4)	
N5	0.9299 (3)	0.5062 (3)	0.5738 (2)	0.0253 (4)	
O1	0.8288 (3)	0.2802 (3)	0.9369 (2)	0.0340 (4)	
O2	0.6617 (3)	0.6912 (3)	1.0508 (2)	0.0403 (4)	
O3	0.8598 (3)	0.9497 (3)	0.7946 (2)	0.0342 (4)	
C1	0.7009 (3)	0.6250 (3)	0.5618 (3)	0.0228 (4)	
H1A	0.829 (4)	0.172 (3)	0.903 (3)	0.034*	
H2A	0.710 (4)	0.784 (3)	0.975 (3)	0.034*	
H3A	1.002 (2)	0.886 (3)	0.754 (3)	0.034*	
H1B	0.922 (4)	0.329 (4)	0.851 (2)	0.034*	
H2B	0.658 (4)	0.707 (4)	1.1563 (18)	0.034*	
H3B	0.797 (4)	1.008 (3)	0.705 (2)	0.034*	

### Atomic displacement parameters (Å^2^)

**Table:**

	*U*^11^	*U*^22^	*U*^33^	*U*^12^	*U*^13^	*U*^23^
Fe1	0.0192 (2)	0.0327 (3)	0.0151 (2)	−0.00481 (17)	−0.00252 (16)	−0.00163 (17)
N1	0.0186 (9)	0.0307 (9)	0.0172 (9)	−0.0021 (7)	−0.0026 (7)	−0.0026 (7)
N2	0.0214 (9)	0.0379 (10)	0.0198 (9)	−0.0024 (8)	−0.0035 (7)	−0.0022 (8)
N3	0.0273 (10)	0.0406 (10)	0.0218 (10)	−0.0020 (8)	−0.0073 (8)	−0.0014 (8)
N4	0.0242 (9)	0.0389 (10)	0.0168 (9)	−0.0043 (8)	−0.0021 (7)	−0.0034 (8)
N5	0.0211 (9)	0.0336 (9)	0.0162 (8)	−0.0053 (7)	−0.0002 (6)	−0.0033 (7)
O1	0.0260 (8)	0.0398 (9)	0.0283 (9)	−0.0044 (7)	−0.0020 (7)	−0.0033 (7)
O2	0.0526 (11)	0.0589 (11)	0.0188 (9)	−0.0333 (9)	−0.0034 (8)	−0.0035 (8)
O3	0.0234 (8)	0.0433 (9)	0.0255 (9)	−0.0014 (7)	−0.0055 (7)	0.0017 (7)
C1	0.0210 (10)	0.0285 (10)	0.0154 (10)	−0.0059 (8)	−0.0023 (8)	−0.0010 (8)

### Geometric parameters (Å, °)

**Table:**

Fe1—O2	2.0868 (15)	N4—C1	1.335 (3)
Fe1—O2^i^	2.0868 (15)	N5—N5^ii^	1.245 (3)
Fe1—O1^i^	2.1081 (16)	N5—C1	1.400 (3)
Fe1—O1	2.1081 (16)	O1—H1A	0.854 (10)
Fe1—N1	2.2474 (16)	O1—H1B	0.851 (10)
Fe1—N1^i^	2.2474 (16)	O2—H2A	0.848 (10)
N1—N2	1.333 (2)	O2—H2B	0.850 (10)
N1—C1	1.338 (3)	O3—H3A	0.852 (10)
N2—N3	1.315 (3)	O3—H3B	0.844 (10)
N3—N4	1.331 (3)		
			
O2—Fe1—O2^i^	180.0	N2—N1—Fe1	119.64 (12)
O2—Fe1—O1^i^	90.39 (7)	C1—N1—Fe1	134.93 (13)
O2^i^—Fe1—O1^i^	89.61 (7)	N3—N2—N1	109.13 (16)
O2—Fe1—O1	89.61 (7)	N2—N3—N4	110.32 (16)
O2^i^—Fe1—O1	90.39 (7)	N3—N4—C1	104.10 (16)
O1^i^—Fe1—O1	180.0	N5^ii^—N5—C1	114.3 (2)
O2—Fe1—N1	91.16 (6)	Fe1—O1—H1A	116.1 (17)
O2^i^—Fe1—N1	88.84 (6)	Fe1—O1—H1B	112.4 (17)
O1^i^—Fe1—N1	89.55 (6)	H1A—O1—H1B	104 (2)
O1—Fe1—N1	90.45 (6)	Fe1—O2—H2A	126.3 (16)
O2—Fe1—N1^i^	88.84 (6)	Fe1—O2—H2B	123.0 (16)
O2^i^—Fe1—N1^i^	91.16 (6)	H2A—O2—H2B	109 (2)
O1^i^—Fe1—N1^i^	90.45 (6)	H3A—O3—H3B	107 (2)
O1—Fe1—N1^i^	89.55 (6)	N4—C1—N1	111.80 (17)
N1—Fe1—N1^i^	180.0	N4—C1—N5	127.64 (18)
N2—N1—C1	104.65 (15)	N1—C1—N5	120.57 (17)

Symmetry codes: (i) −*x*+1, −*y*+1, −*z*+2; (ii) −*x*+2, −*y*+1, −*z*+1.

### Hydrogen-bond geometry (Å, °)

**Table:**

*D*—H···*A*	*D*—H	H···*A*	*D*···*A*	*D*—H···*A*
O2—H2A···O3	0.85 (1)	1.86 (1)	2.699 (2)	170 (2)
O1—H1A···O3^iii^	0.85 (1)	1.87 (1)	2.715 (2)	171 (3)
O1—H1B···N5	0.85 (1)	2.23 (2)	2.926 (2)	139 (2)
O3—H3B···N3^iv^	0.84 (1)	2.01 (1)	2.839 (2)	169 (2)
O3—H3A···N2^v^	0.85 (1)	2.00 (1)	2.843 (2)	173 (2)
O2—H2B···N3^vi^	0.85 (1)	2.69 (2)	3.439 (2)	148 (2)
O2—H2B···N4^vi^	0.85 (1)	1.99 (1)	2.840 (2)	173 (2)

Symmetry codes: (iii) *x*, *y*−1, *z*; (iv) −*x*+1, −*y*+2, −*z*+1; (v) *x*+1, *y*, *z*; (vi) *x*, *y*, *z*+1.

## References

[bb1] Bruker (2002). *SADABS*, *SAINT* and *SMART* Bruker AXS Inc., Madison, Wisconsin, USA.

[bb2] Hammerl, A., Gerhard, H., Klapötke, T. M., Mayer, P., Nöth, H., Piotrowski, H. & Warchhold, M. (2002). *Eur. J. Inorg. Chem.* pp. 834–845.

[bb3] Hammerl, A., Klapötke, T. M., Nöth, H. & Warchhold, M. (2001). *Inorg. Chem.* **40**, 3570–3575. 10.1021/ic010063y 11421707

[bb4] Jiao, B. J., Chen, S. P., Zhao, F. Q., Hu, R. Z. & Gao, S. L. (2007). *J. Hazard. Mater.* **142**, 550–554. 10.1016/j.jhazmat.2006.07.066 16978770

[bb5] Sheldrick, G. M. (2008). *Acta Cryst.* A**64**, 112–122. 10.1107/S0108767307043930 18156677

